# REVOLUTIONIZING DIGITAL ENGAGEMENT: EFFECTS OF AN APP ENABLING PERSONS WITH DEMENTIA TO LEAD ACTIVITIES FOR PEERS

**DOI:** 10.1093/geroni/igad104.3744

**Published:** 2023-12-21

**Authors:** Michael Skrajner, Gregg Gorzelle, John Zeisel, Drew Walker

**Affiliations:** Hopeful Aging, Bolivar, Ohio, United States; Hopeful Aging, Hiram, Ohio, United States; Hopeful Aging, Winchester, Massachusetts, United States; Hopeful Aging, Winchester, Massachusetts, United States

## Abstract

A Cluster Randomized Trial was conducted to evaluate the effects of an app that enables long-term care (LTC) residents with early-stage dementia to lead activities for their peers—i.e., other persons living with dementia (PLWD). A total of 72 PLWD completed the study, including 35 Control Group PLWD (Standard Programming) and 37 Experimental Group PLWD (Resident-Led Programming). Resident-Leaders exhibited 99% overall adherence to the steps required to lead activities and required minimal help from staff. A series of repeated measures ANCOVAs, controlling for Mini Mental State Examination (MMSE) scores, were conducted to examine proximal (immediate) and distal (longer-term) effects of the intervention. Regarding proximal effects, there were significantly greater increases from baseline to treatment in Constructive Engagement (p<.01), Passive Engagement (p<.01), and Pleasure (p<.01), as well as significantly greater decreases in Distracted Engagement (p<.01) and Passive Engagement (p<.01), for the Experimental Group, as compared to the Control Group. Regarding distal effects, there were no significant differences between trends observed by Control and Experimental Group residents from baseline to post-treatment for neuropsychiatric symptoms, agitation, depression, or quality of life. However, for a subsample of the Experimental Group (PLWD who resided in Assisted Living), there was a significant decrease in depressive symptoms (p<.05) and increase in quality of life (p<.01). Data suggest that this is a highly engaging intervention that enables PLWD to lead activities for their peers. The intervention has its greatest impact during the session, when positive forms of engagement/affect are increased, and negative forms of engagement are decreased.

